# A Coupling Framework for Multi-Domain Modelling and Multi-Physics Simulations

**DOI:** 10.3390/e23060758

**Published:** 2021-06-16

**Authors:** Dario Amirante, Vlad Ganine, Nicholas J. Hills, Paolo Adami

**Affiliations:** 1 Thermo-Fluid Systems UTC, University of Surrey, Guildford GU2 7XH, UK; v.ganine@surrey.ac.uk (V.G.); n.hills@surrey.ac.uk (N.J.H.); 2 Rolls-Royce Deutschland, Eschenweg 11, 15827 Blankenfelde-Mahlow, Germany; paolo.adami2@Rolls-Royce.com

**Keywords:** code coupling, conjugate heat transfer, overset

## Abstract

This paper describes a coupling framework for parallel execution of different solvers for multi-physics and multi-domain simulations with an arbitrary number of adjacent zones connected by different physical or overlapping interfaces. The coupling architecture is based on the execution of several instances of the same coupling code and relies on the use of smart edges (i.e., separate processes) dedicated to managing the exchange of information between two adjacent regions. The collection of solvers and coupling sessions forms a flexible and modular system, where the data exchange is handled by independent servers that are dedicated to a single interface connecting two solvers’ sessions. Accuracy and performance of the strategy is considered for turbomachinery applications involving Conjugate Heat Transfer (CHT) analysis and Sliding Plane (SP) interfaces.

## 1. Introduction

Future simulation technologies will increasingly rely on the capability to perform flexible coupling between existing solvers. Multi-physics problems, including Fluid–Structure (FS) interactions, moving parts, and Conjugate Heat Transfer (CHT), have recently become common within the industry, and several commercial packages offer integrated systems to solve them. Coupling procedures are also widely used for standalone CFD computations: in turbomachinery and rotorcraft applications, for example, the computational domain is divided into regions where the flow equations are solved in different frames of reference to account for the relative motion of objects. Overset (Chimera) methods or sliding plane techniques are usually adopted to couple the solutions between the zones [1,2,3].

More generally, there are classes of flow that, due to their inherent hybrid nature (e.g., different Reynolds or Mach numbers), can be better resolved using locally specialised flow solvers or computational settings; a typical example in a turbomachinery application is the incompressible reacting flow from a combustion chamber coupled to a compressible and (usually) in-equilibrium mixture flowing trough heavily cooled turbine inlet guide vanes [4,5].

A class of methods gaining popularity is that of segregated (also referred to as zonal) methods, where hybrid RANS/LES modelling closures are coupled together. In this case, the domain is split using the idea that the best turbulence closure is more effective at different zones of the flow; the interfaces must guarantee both the physical and statistical compatibilities between the diverse modelling approximations [5,6,7].

The growing importance of similar requirements, combined with the modularity available in a modern simulation environment, naturally suggest the need for some capability allowing for coupling of these individual physical modules, discretisation techniques, or mixed fidelity methods. Good examples of this approach are the CEDRE software package [8] developed at ONERA and the zonal flow solver of Schröder and his group [9,10].

A common issue with code coupling methods is the need to combine flexibility and accuracy with simplicity and parallel performance. Following Larson et al. [11], a parallel coupled model can be schematised by a net of vertices representing individual solvers that interact through connecting edges. This is a decentralised model suitable for the parallelisation of heterogeneous complex systems where the individual solvers are managed through input information from their neighbors. If the information is available, they are able to proceed, while they have to wait if this is not the case: from the parallel performance point of view, the system therefore behaves in the same way as the parallel processes of a monolithic code.

In order to maintain full scalability of each solver, the communications along the edges should remain a distributed process. In a parallel environment with distributed memory, this requires peer-to-peer communications, i.e., direct data transfer among the adjacent processes handling the various interfaces. Popular coupling libraries such as preCISE [12], MpCCI [13], or the Multiscale Universal Interface of Tang et al. [14] follow this strategy. However, direct communications are less efficient (and more difficult to handle) for interfaces with moving boundaries because, in this case, the connectivity patterns need to be dynamically updated.

More generally, one has to consider the case where the mutual interaction between two distinct solvers involves a significant amount of computational work. For example, operations such as searching algorithms, interpolating, filtering, or reading from an external database may all be required by the coupling. In this case, the scalability of the whole system cannot be achieved by balancing only the discretisation work load of each solver. It is also necessary to consider the processing of the data at the interfaces and the impact that the updating strategy has on the convergence of each single domain. Ganine et al. [15] illustrated this scenario for an unsteady simulation involving two zones in a turbine stage: in their example, a performance improvement in the coupled simulation was achieved by dedicating a set of intermediary processors to exclusively perform the search and interpolation operations needed to exchange across the moving interface. The benefit here stems from the fact that the interpolation can be evenly distributed among the coupling processors, while some of these operations can be overlapped with the work carried out at the same time by the two solvers [15].

**Figure 1:**
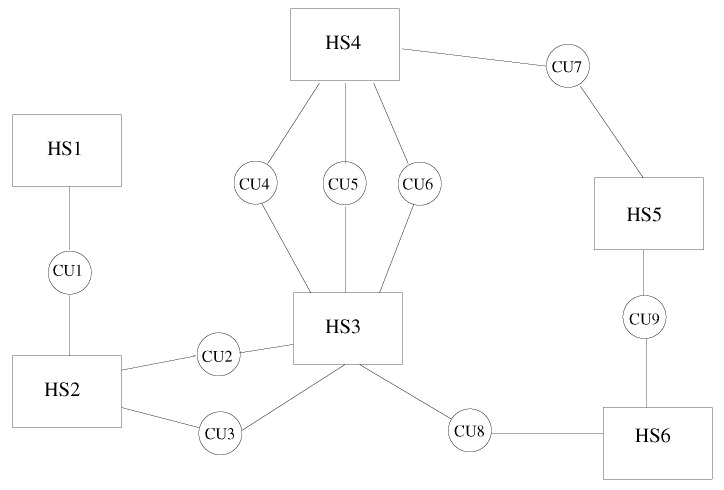
Coupling architecture of JMxx. A Coupler Unit (CU) is associated with only one interface between two Hydra Sessions (HS). In this example, HS1 and HS2 share one interface HS3 and HS4 share three interfaces, etc.

In this paper, we describe a coupling architecture that combines the decentralised model of Larson et al. [11] with the client-server scheme of Ganine et al. [15]. The aim is to build a unified framework able to preserve the modular flexibility of the former and the performance of the latter. The context of our work is the Rolls-Royce proprietary suite of solvers Hydra [3,16,17]. Hydra is an unstructured code designed for turbomachinery applications, equipped with a range of validated modules for CFD and thermal analysis. Inspired by the concept of “smart edges” discussed by Hoekstra et al. [18], also known as “smart interface methodology” [19], a coupling framework (here, referred to as JMxx) was developed following the schematic of Figure 1. The whole system is decomposed in a number of solver Hydra Sessions (HS) mutually interacting through individual Coupler Units (CU). The Hydra Sessions are different models running on distinct meshes that cover adjacent or overlapping zones of the physical space. A Coupler Unit is a set of one or more processes dedicated to carrying out specific coupling procedures between two Solver Sessions. The framework is general and can allow for coupling of different solvers, but for the purpose of the present work, the discussion focuses on the use of Hydra as solver.

A distinctive feature of JMxx is that a single Coupler Unit manages only one interface, namely the interface (or one of the interfaces) shared by the two associated Hydra Sessions. In case of multiple interfaces between two Hydra Sessions, then multiple Coupler Units are required, one for each interface. As result of this feature, each Coupler Unit works exclusively on data related to two “attached” Hydra Sessions. Data structure, communication pattern, and connectivity are therefore all independent from any other interface and not affected by the complexity of the overall system. Perhaps more importantly, this direct correspondence between interfaces and Coupler Units introduces a first coarse level of parallelisation into the topology of the system thanks to the specification of the separate interfaces. In applications where the relative motion between the zones remains confined in regions known a priori, both communications and spatial global search can be easily made scalable by appropriate definition of the interfaces.

The paper is organised as follows: a short description of Hydra is given in Section 2. Only the necessary details are given, while the main focus is on the general infrastructure of JMxx that is detailed in Section 3. The results are presented in Section 4, Section 5 and Section 6. First, the methodology is validated for a CHT analysis of a forced convection flow. A model of the internal air system of a low pressure turbine is then used to demonstrate the application of the method to a more complex system. Finally, the scalability characteristics of JMxx are examined for a model problem, and the parallel performance is discussed for a test case involving a sliding plane interface.

## 2. Hydra Solvers

Hydra consists of a suite of fluid and thermal unstructured solvers developed collaboratively by Rolls-Royce plc and its university partners. All solvers are finite-volumes and use an edge-based data structure generated by the same preprocessing tool. The variables are stored at the cell vertices, and the control volume is defined by the “median-dual” around each mesh node [16]. Hydra solves the compressible Navier–Stokes equations and has a number of popular turbulence models for RANS and LES. The spatial discretisation uses the approximate Riemann solver of Roe for the evaluation of the convective fluxes. A second order of accuracy for the convective fluxes is achieved by introducing pseudo-Laplacian operators in the upwind contribution of the Roe dissipative flux, as detailed in the work of Moinier [16]. The gradients are computed with a Green–Gauss method on the mesh nodes, and a second-order finite-volume discretisation for the viscous fluxes is obtained by averaging the gradients at the cells interface. In RANS computations, the flow equations are iterated towards steady state using a Runge–Kutta (RK) *m*-stage method. Convergence is accelerated by incorporating the 5-stage RK scheme within an edge-collapsing multigrid algorithm [20]; alternatively, the implicit formulation of Swanson et al. [21,22] can be used for the three-stage RK scheme executed on a single grid level. The thermal solver inherits most of the features from the flow solver. The unsteady equation for solid heat conduction is iterated towards steady-state using the five-stage RK scheme with multigrid. Simplified versions of the subroutines used in the flow solver are adopted to compute the viscous fluxes and to evaluate the residuals for the heat conduction equation.

JMxx was designed to simulate complex configurations with several fluid and/or solid zones, individually solved by Hydra and coupled through appropriate interfaces. A Conjugate Heat Transfer (CHT) interface is used to enforce thermal coupling between fluid and solid zones [13,23,24]. The wall temperature retrieved from the solid domain is prescribed as a boundary condition for the fluid domain, whereas the heat flux retrieved from the fluid side is applied on the boundaries of the solid domain. An under-relaxation parameter (applied to the boundary condition changes) is used to ensure stability [25]. The interfaces between solid regions in contact are treated following the same method of CHT interfaces, enforcing the continuity of temperature and heat flux. In this work, they are referred to as Thermal Contact (THC) interfaces.

The simulations with multiple fluid zones use mixing planes, sliding planes, or overset interfaces. Mixing planes are a standard type of interface adopted between stationary and rotating rows in steady RANS of turbomachinery applications. Unsteady computations require sliding planes to account for the relative movement between rotor and stator. In Hydra, these were implemented following the method of Blades et al. [15,26]. A layer of halo nodes on each side of the interface form a one cell overlap with the adjacent zone. The two solutions are updated in a different frame of reference, and the flow variables of one zone are interpolated, after appropriate rotation, to set the flow variables on the halo nodes of the opposite zone. The method requires a search algorithm at any time step to identify the donor element for each target node.

The overset interfaces (OSET) are based on the Chimera method [1,27,28] and can be used for a variety of reasons, such as simplifying meshing around complex geometries, increasing resolution locally, or treating moving objects. The computational domain is discretised with overlapping grids. In general, one of the two grids (the “overset mesh”) is fully embedded into the other (the “host mesh”), and the difference between the two defines a hole within the host mesh. The grid points of the host mesh that lie within the hole are excluded from the computation, and the grid points surrounding the hole form an artificial boundary (the “fringe”), where the flow variables are interpolated.

In JMxx, each zone corresponds to a Hydra Session, any inter-grid communication is regarded as an interface, and the data transfer between the grids is handled by dedicated Coupler Units.

## 3. Coupling Framework

**Figure 2:**
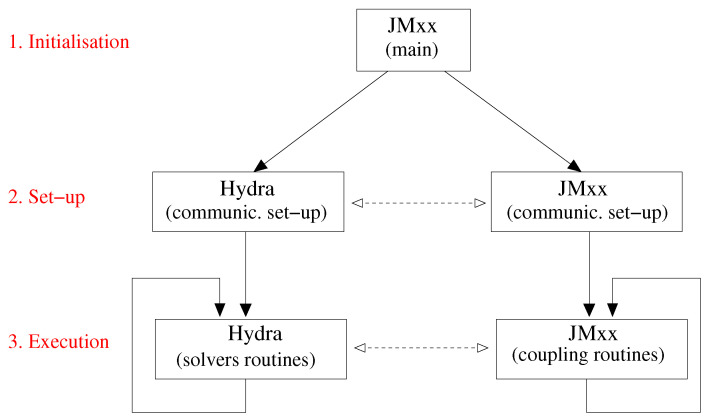
Coupling framework organisation with the three distinct phases: initialisation, set-up, and execution.

The coupling framework is organised as shown in Figure 2. Two distinct applications, JMxx and Hydra, operate in three subsequent phases. JMxx starts the program and, during an initialisation phase, prepares the parallel environment defining the subgroups and launching the execution of Hydra Sessions (HSs) and Coupler Units (CUs). At the end of this step, a set-up phase establishes the communications between HSs and CUs. This is followed by the effective execution phase, during which Hydra and JMxx carry out their internal iterations and exchange data throughout. The program is launched with the standard SPMD syntax

mpirun -npnproc jmxx_exe

where jmxx_exe is the executable obtained linking JMxx against Hydra, previously compiled with its entry point converted into subroutine. The next subsections describe the initialisation phase; the communications; and the execution phase, emphasising, in particular, the infrastructure and the communicational scheduling.

### 3.1. Initialisation Phase

The data structure of JMxx is based on two derived data types: a Coupler Table and a Hydra Table. Typical sections of these structures are shown in Table 1. It can be noticed that most of the records in the Coupler Table have a dichotomous structure. This reflects the paradigm that one Coupler Unit is always linked with two Hydra Sessions. On the other hand, an Hydra Session may have an arbitrary number of interfaces and, therefore, may be connected to an arbitrary number of CUs. Thus, most of the records in the Hydra Table are variables sectioned according to the number of coupled interfaces (*ncoupl*). All of the required information, including ranks, sizes, models options, memory addresses, etc., are organised in the same way.

JMxx reads the number of Hydra Sessions $\left(n_{HS}\right)$ and the number of Coupler Units $\left(n_{CU}\right)$ involved in the computation from an input file. Immediately afterwards, *all* processes allocate memory to store the data tables. More precisely, every process keeps $n_{HS}$ Hydra Tables and $n_{CU}$ Coupler Tables in memory. At this point, JMxx reads the number of cores dedicated to each HS and to each CU from the input file along with the interconnections between the various sessions and units. This information defines the topology of the coupled system and is stored in the appropriate records of the Hydra and Coupler Tables.

Now, the program creates $n_{HS}+n_{CU}$ local communicators for individual subsets with the prescribed number of cores. At the end of this step, every process remains associated with a group rank, a code identifier, and a session identifier. The code identifier is used to divert the various processes towards the Hydra entry point or towards the inner routines of JMxx. Hydra is invoked passing the local communicators that have to replace the MPI_COMM_WORLD for the internal communications. This is achieved by initialising the parallel environment in Hydra by MPI_Comm_dup instead of MPI_Init. Following this procedure, one ends up with $n_{HS}$ instances of Hydra, and $n_{CU}$ instances of the JMxx coupling routines, obtaining an effect similar to what the mpirun command would produce if executed in MPMD mode for distinct programs. This step marks the end of the initialisation phase.

Throughout the rest of the computation, the session identifier is used by each process to single out the Hydra Table or the Coupler Table pertaining to the partition. Such an organisation makes programming simple and intuitive, as can be seen from the two pieces of code reported in Algorithms 1 and 2. In the first example, a CU process accesses the ranks of the processes handling the two connected HSs, obtains the sizes of the corresponding interfaces, and sets pointers to the memory address of node coordinates. The second example illustrates the dual operation performed by a HS process, with the external loop executed over the number of interfaces (ncoupl) defined for the Hydra Session.
**Algorithm 1** Access to data from Coupler Unit (CU) processesiset=id_set(my_rank)        ▹Session id of the CU process**for**im=1,2**do**                   ▹ Loop over the two connected HSs    nproc=ctable(iset).hproc(im)        ▹ Number of processes handling this HS    **for** ip=1,nproc **do**        rank_to=ctable(iset).rank_h(ip,im)     ▹ Rank of processes handling this HS        ntnode=ctable(iset).ntnode(ip,im)            ▹ Number of target nodes        xtarget→ctable(iset).p_xtarget(ip,im)        ▹ Set pointer to target nodes    **end for****end for**

**Algorithm 2** Access to data from Hydra Session (HS) processes
iset=id_set(my_rank)                   ▹ Session id of the HS processncoupl=htable(iset).ncoupl                 ▹ Number of connected CUs**for**ic=1,ncoupl**do**                   ▹ Loop over the connected CUs    nproc=htable(iset).cproc(ic)         ▹ Number of processes handling this CU    **for** ip=1,nproc **do**        rank_to=htable(iset).rank_c(ip,ic)         ▹ Rank of processes handling this CU        ntnode=htable(iset).ntnode(ip,ic)             ▹ Number of target nodes        xtarget→htable(iset).p_xtarget(ip,ic)         ▹ Set pointer to target nodes    **end for**
**end for**



### 3.2. Communications

JMxx uses an element containment test for the interpolation. Therefore, CU processes need to receive nodal coordinates and connectivity arrays on both sides of their interfaces. After the mesh has been partitioned within Hydra, the HS processes form a list of local target nodes (local in the sense of pertaining to the partition), a list of local source nodes, and a list of mesh elements connecting the local source nodes. In the case of an overset interface, the source elements are grid cells, whereas for interfaces defined on a surface, such as sliding planes, they are boundary faces. Note that source and target nodes may or may not point to the same geometric entity, depending on the interface type. In any case, they form separate lists because the way in which they are accessed is different. The local lists with nodes and elements constitute the mesh topology relative to the portion of interface owned by each partition. The lists are sent to the connected CU processes which, in turn, assemble the various patches forming two global lists of target nodes, two global lists of source nodes, and two global lists of source elements. The global lists define the entire mesh of the interface.

**Figure 3:**
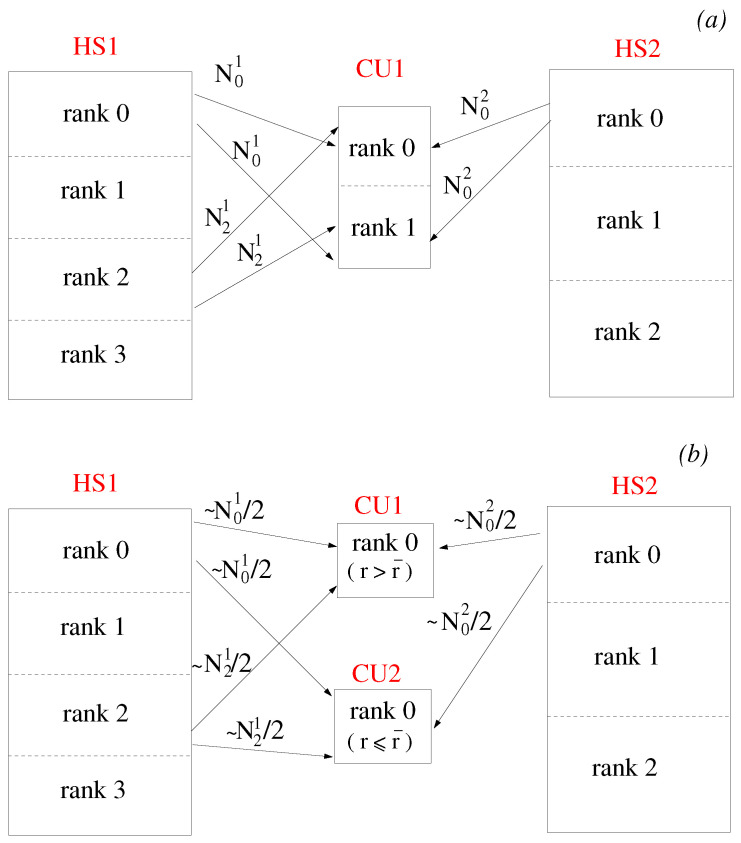
Parallelisation strategies in JMxx. (**a**) One Coupler Unit with two MPI ranks. (**b**) Two Coupler Units with one MPI rank each. $N_{i}^{j}$ is the number of mesh nodes belonging to rank *i* of Hydra Session *j* that lie on the interface.

All processes of a single Coupler Unit keep the whole mesh of the associated interface in memory. When performing the interpolation, the search algorithm is carried out on the whole pool of source elements while the target nodes are equally distributed among the various CU processes. An example of this architecture is shown in Figure 3a. A Coupler Unit with two MPI processes handles the interface between HS1 and HS2. Each Hydra partition with mesh nodes lying on the interface (rank = 0 and rank = 2 for HS1, and rank = 0 for HS2) sends the same set of data to both rank = 0 and rank = 1 of the Coupler Unit. Each process of the Coupler Unit interpolates half of the total number of target nodes. Clearly, this is not an optimal solution, because although searching and interpolation scale linearly with the number of CU processes, the amount of data received by each CU process remains constant.

A more scalable approach can be easily obtained in JMxx by reproducing an effect similar to the coarse level bounding box discussed by Sitaraman et al. [29]. The main idea is based on the fact that the communication paths are constructed separately depending on the prescribed topology. If each interface is uniquely associated with a Coupler Unit, the communications can be parallelised by appropriately splitting the interface between two Hydra Sessions. The Hydra processes access the source nodes by looping over the mesh elements; the corresponding data are packed and scattered among the CUs depending on a membership relation between mesh element and interfaces. Membership of an interface can be made subordinate to geometric constraints specified by the user in the input file. For example, in Figure 3b, the interface is split in two radial bands, lower and upper, and a single Coupler Unit with one process is dedicated to each band. In this case, the HS processes send to the Coupler Units only the nodes located above or below a certain radius $\bar{r}$. Thus, assuming a well-balanced choice of $\bar{r}$, the number of data received by each CU process is halved compared to the case of Figure 3a. In this way, the overhead for both interpolation and communications is expected to scale linearly whereas the work load for the search algorithm (assuming a brute force method) scales quadratically. Compared to peer-to-peer communications strategies, this approach keeps the program simple and minimally invasive because there is no need to map the internal partitioning of one Hydra Session into the other: the Hydra processes just need to know which Coupler Unit is dedicated to which band and can ignore the distribution of source nodes in the coupled Hydra Sessions.

It is important to remark that the same concept can be applied, identically, to an interface consisting of a volume portion. The only limitation for the method is that the relative movement between the grid nodes must be confined to the same spatial regions, so that the communication paths between HSs and CUs do not change. Cases with arbitrary motion of the interfaces are therefore out of scope.

### 3.3. Execution Phase

Algorithm 3 describes the sequence of operations performed by Hydra processes during the execution phase. The steps reported in Algorithm 3 are representative of a generic pseudo-time marching scheme. There is an external loop over the number of iterations to perform (ncycle), an intermediate loop defined by the multigrid cycle, and an inner loop representing the Runge–Kutta stages. When running with the explicit scheme, the multigrid cycle consists of a few smoothing iterations (generally 1 or 2) performed by the RK5 method on each grid level. For the semi-implicit scheme, a cycle corresponds to a number of Richardson iterations (≤5) performed within the RK3 scheme on the finest grid [22]. For unsteady computations, the operations described above are nested into a further loop over the physical time steps. In this case, ncycle assumes the meaning of subiterations of a Dual Time Stepping scheme, and an additional communication point occurs after the solution update. In the analysis that follows, we focus on steady computations because, in this case, there is a greater degree of freedom in choosing the parameters that define the coupling.
**Algorithm 3** Sequence of operations of Hydra Session (HS) processes**for**n=1,ncycle**do**     Post communication requests    **while** cycle *n* is not complete **do**        **for** ns=1,nstage(RK) **do**            Compute residuals           **if** implicit **then**                Perform Richardson iterations           **end if**            Update        **end for**        **if** explicit **then**            Prolong or Restrict solution        **end if**    **end while****end for**

A communication request from a HS process consists of the following steps:

1. Loop over the interfaces to open communications for receiving data from all connected CU processes (MPI_Irecv).
2. Loop over the interfaces to send data to all connected CU processes (MPI_Isend).
3. Wait for the receive operations to be completed (MPI_Waitall).
4. Loop over the interfaces to unpack the data received.

When dealing with systems integrating different physics or systems where interfaces of different nature coexist, it is key to leave the user with the possibility of prescribing individual coupling frequencies on both sides of each Coupler Unit. When parsing a specific interface, at steps 1 and 2 described above, the corresponding MPI_Irecv and MPI_Isend operations are skipped if the iteration counter does not match the coupling frequency specified for that interface. This approach provides great flexibility but requires some care to avoid deadlock in coupling topologies containing a cycle.

**Figure 4:**
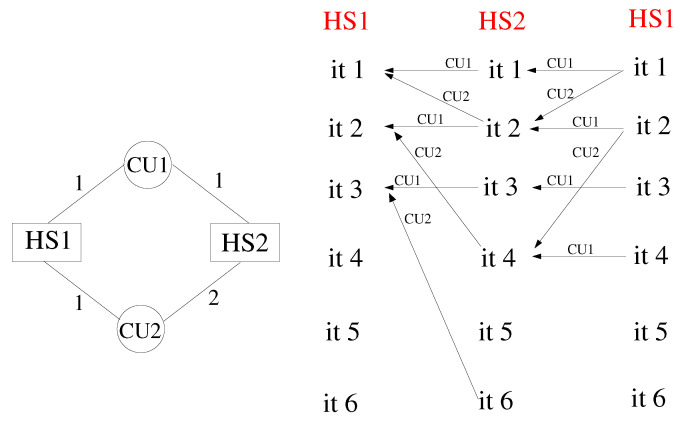
Example of cyclic JMxx model with deadlock and corresponding dependency patterns.

**Figure 5:**
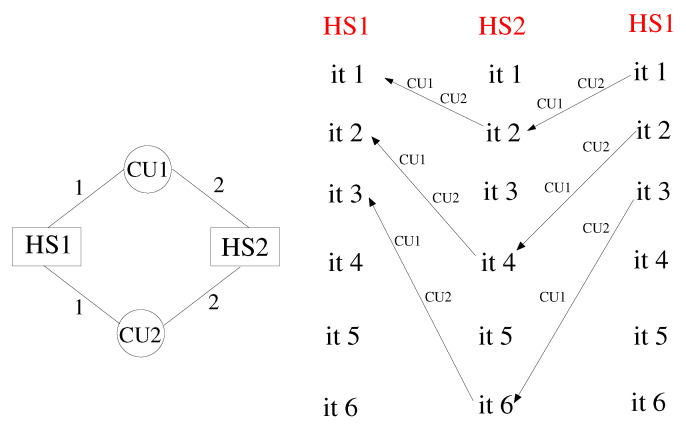
Example of cyclic JMxx model without deadlock and corresponding dependency patterns.

Consider the simple cycle of Figure 4, with 2 HSs and 2 CUs. The numbers reported on the edges denote the coupling frequency on each side of the two interfaces. According to the scheme, HS1 processes post a communication request every iteration for both CU1 and CU2. Likewise, HS2 processes post a communication request every single iteration for CU1 and every two iterations for CU2. This means that iteration *n* of HS1 can be performed only when HS2 arrives at iteration 2n, while iteration *n* of HS2 requires HS1 to be at iteration *n*. The dependency is illustrated in the patterns of Figure 4, reporting the CUs across which the dataflow takes place. In this case, a deadlock occurs at the second iteration. At this stage, in fact, HS1 waits for data from CU2, which cannot arrive, because HS2 cannot execute iteration 3. In order to correct the model, it is necessary to recover a synchronised mutual dependency. More formally, if iteration *i* of HS1 depends on iteration *j* of HS2, then iteration *j* of HS2 must depend on iteration *i* of HS1. An example of a synchronised cycle is shown in Figure 5, but this is not the only choice, and in Appendix A, we report simple guidelines that can be followed to avoid deadlock, whatever the topology of the system.

The operations performed by Coupler Unit processes are listed in Algorithm 4. The set of instructions depends on the type of interface associated with the CU (variable *ctype* in Algorithm 4). For example, in a CHT interface, there is no relative motion and the search algorithm is executed only once. Conversely, in unsteady simulations with sliding planes, the search has to be repeated for every time step. The algorithm is invoked before entering the internal loop on the number of Dual Time Step subiterations (ncycle), and after that non-blocking receive messages have opened the communications. In this way, the search, which does *not* require updated data from HSs, is initiated while the HS processes are busy in the last subiteration of previous time step and then overlapped with the subsequent communication phase.
**Algorithm 4** Sequence of operations of Coupler Unit (CU) processesiset=id_set(my_rank)               ▹ Session id of the CU processctype=ctable(iset).ctype                  ▹ Type of interface **if**(ctype=slidingplane)**then**    **while** true **do**                     ▹ Loop until completion         Open communications to receive data        Search algorithm                 ▹ Search for this time step        **for** i=1,ncycle **do**              ▹ Loop over internal subiterations            **if** (i≠1) open communications to receive data            Wait until receive is completed            Interpolate data on target nodes            Send interpolated data        **end for**    **end while****end if** **if**(ctype=CHTinterface)**then**     Search algorithm                ▹ Search is performed only once    **while** true **do**                      ▹ Loop until completion         Receive data on source nodes         Interpolate data on target nodes         Send interpolated data    **end while****end if** **if**(ctype=anyothertypeofinterface)**then**    ...                     ▹ Organise operations as appropriate**end if**

## 4. Forced Convection on a Conductive Solid Square

**Figure 6:**
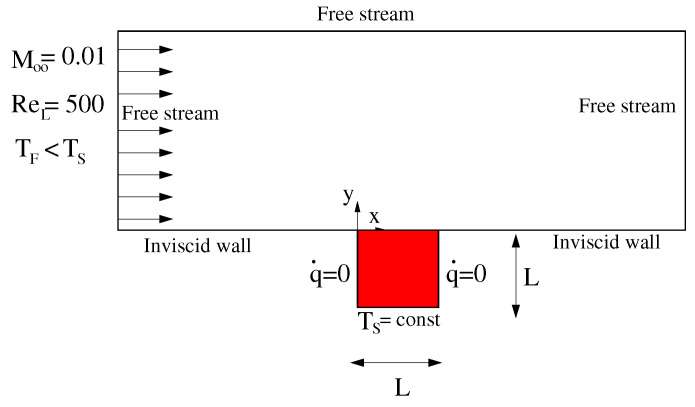
Problem definition for the forced convection flow on a conductive solid block.

The first test case considered is the conjugate heat transfer analysis of forced convection flow over a conducting solid square. The physical model is shown in Figure 6. A laminar, incompressible flow with a uniform temperature of $T_{F}$ moves over a square block of solid with thermal conductivity $k_{S}$. The lower side of the solid has a constant temperature $T_{S}>T_{F}$, while the two sides normal to the flow direction are adiabatic walls. Free stream conditions are prescribed to match a Mach number $M_{\infty }=0.01$ and a Reynolds number based on the square length *L* equal to $Re_{\infty }=500$. With the further specification of the Prandtl number Pr and the thermal conductivity ratio between solid and fluid $\lambda =k_{F}/k_{S}$, the problem is defined in terms of nondimensional temperature $T^{*}=\left(T-T_{F}\right)/\left(T_{S}-T_{F}\right)$. Here, we consider the case studied by Vynnycky et al. [30] with Pr=100 and λ=20.

### 4.1. Model Set-Up

**Figure 7:**
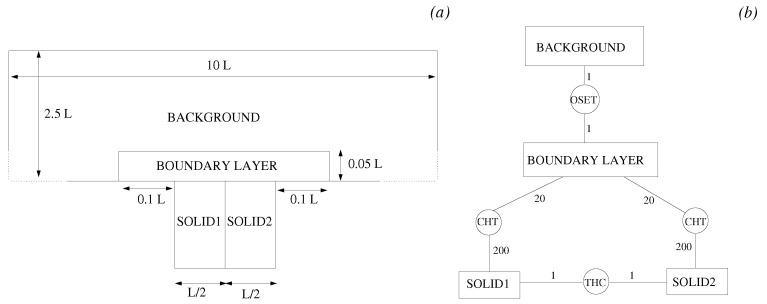
JMxx model adopted for the forced convection conjugate problem. (**a**) Definition of fluid/solid domains. (**b**) Interconnections between Hydra Sessions and Coupler Units.

The JMxx model adopted for this case study was deliberately overcomplicated for the purpose of validation. The fluid region is divided into a boundary layer zone enclosed into a coarser background mesh, with the latter extending from the wet surface of the solid to the free stream (Figure 7). The conductive solid is formed by two adjacent regions with equal thermal conductivity. Each solid region is coupled to the boundary layer fluid zone via CHT interfaces, whereas the interconnection between the two solid components occurs through a Thermal Contact interface. In the sketch of Figure 7b the coupling frequencies on both sides of each Coupler Unit are also reported. In general, it is convenient to keep the coupling frequency low for fluid–fluid and solid–solid interfaces, to avoid excessive decoupling of the solutions. For CHT interfaces, this constraint is less stringent, and the communication requests on the solid side can be posted after a large number of iterations in order to accelerate convergence.

**Figure 8:**
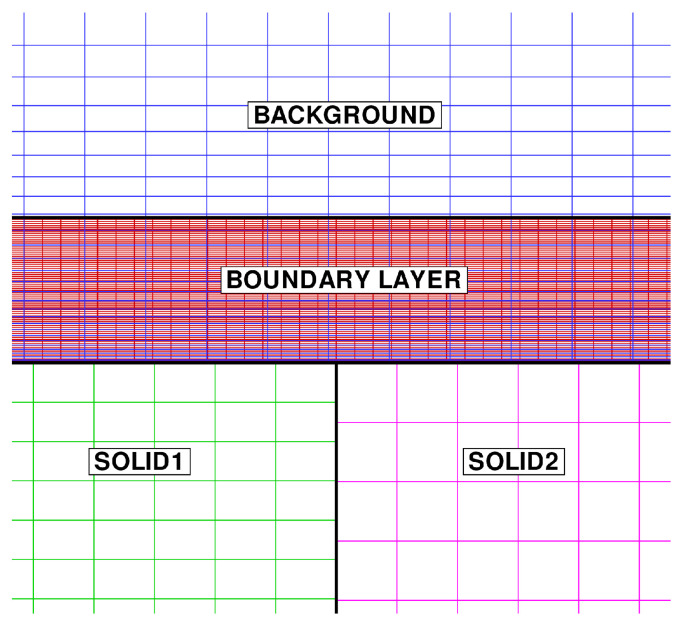
Forced convection conjugate problem. Close-up view of the fluid/solid meshes.

As shown in Figure 8, a substantially different mesh resolution is employed for the various domains. In the region where the background mesh and the boundary layer mesh overlap, the axial and radial grid spacing of the background mesh are about three and seven times larger, respectively, than in the boundary layer mesh. The cell size of the two solid models is similar, but the grid point distribution along the THC interface is not conformal.

Computational resources are allocated by the user depending on the complexity of each model and based on the selected coupling frequency. Denoting by $W_{i}$ a measure of the workload (per rank) associated with the *i*th Hydra Session, a Coupler Unit is balanced if

(1) $$\begin{array}{c} W_{i}\times f_{i}=W_{j}\times f_{j} \end{array}$$

in which $f_{i}$ and $f_{j}$ are the coupling frequencies between the Coupler Unit and the attached Hydra Sessions. Load balancing requires the above condition to be fulfilled for all Coupler Units. Note that an appropriate distribution of the resources along with a pertinent selection of the coupling frequencies can be exploited to speed-up the convergence of the coupled system. In this paper, we focus more on detailing the architecture of JMxx, providing the relevant validation. Hence, this type of analysis is not reported and the reader is referred to [31] for a demonstration of this feature.

### 4.2. Results

**Figure 9:**
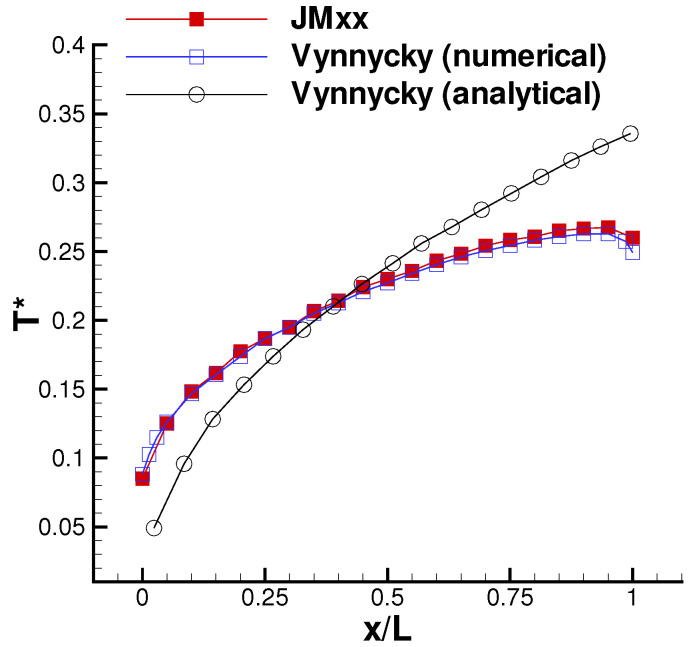
Forced convection conjugate problem. Computed wall temperature.

**Figure 10:**
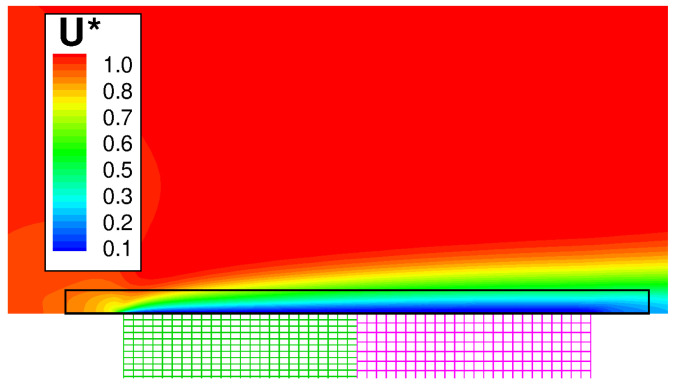
Forced convection conjugate problem. Axial velocity contour plot $U^{*}=U/U_{\infty }$.

**Figure 11:**
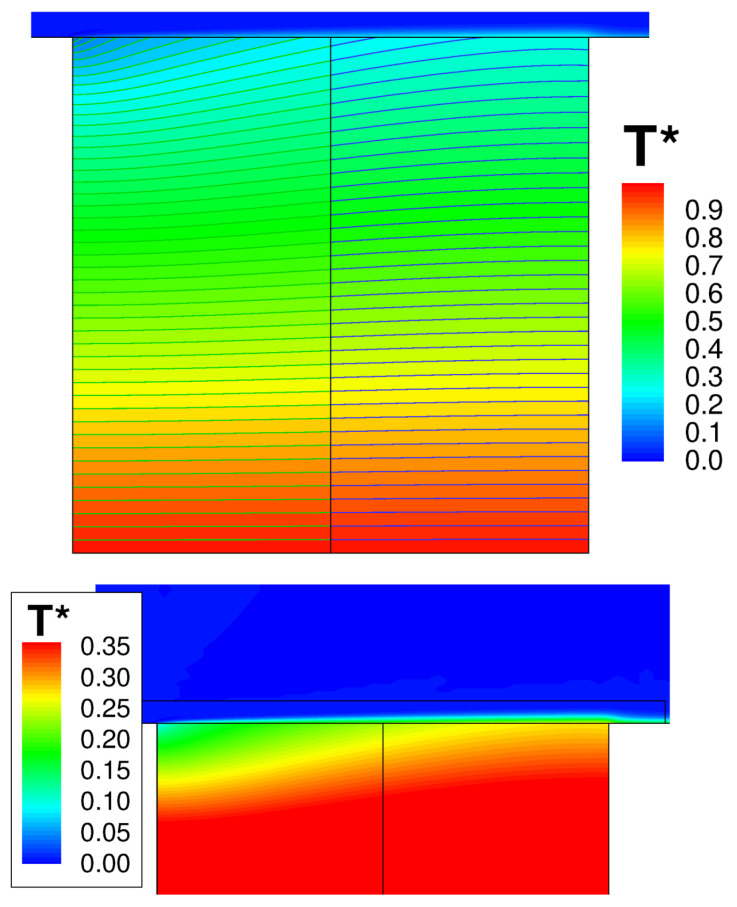
Forced convection conjugate problem. Temperature contour plots. Top: global view of the solid. Bottom: close-up view of the solid–fluid interface.

Figure 9 shows the wall temperature computed along the outer surface of the solid. The result is in excellent agreement with the numerical solution of Vynnycky et al. [30], obtained using a finite difference code. A contour plot of the axial velocity is shown in Figure 10. It should be noted that the domain of the background mesh penetrates a certain “cutting distance” (specified by the user) within the domain of the boundary layer. The cutting distance selected for this case covers a layer of four cells in the background mesh. Thus, there exists a narrow region where the two solutions overlap, and these are both visualised by the graphic solver. The absence of blur in the figure denotes that the two coexisting solutions converge towards the same flow field. Figure 11 shows the computed temperature field. Even in this case, the smooth behaviour of the isotherms across the THC interface confirms the good quality of the coupled solution. Note that, with a Prandtl number equal to Pr=100, the thermal boundary layer is much thinner than the momentum boundary layer and remains resolved withing a few mesh nodes of the fluid BOUNDARY LAYER zone.

## 5. Secondary Air System of a Low-Pressure Turbine

**Figure 12:**
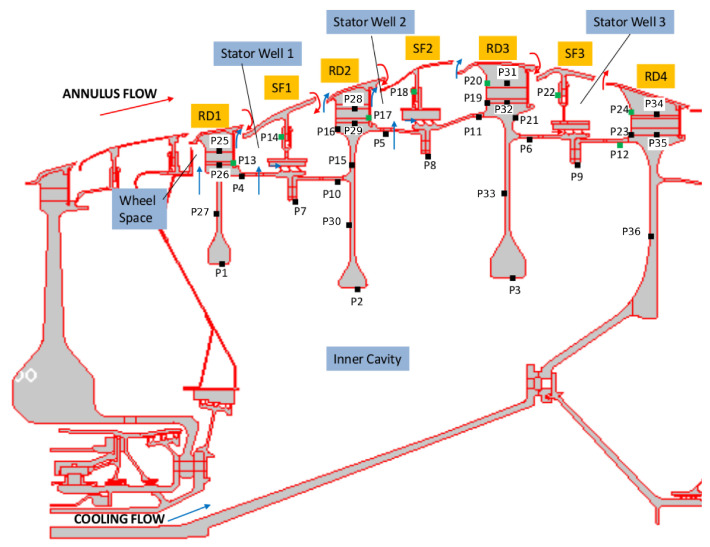
Secondary air system of a low-pressure turbine and definition of control points (Pno.). Black symbols correspond to thermocouple positions. Green symbols are additional control points selected for the analysis.

Figure 12 shows the secondary air system in the low-pressure turbine of an aircraft engine. Various arrows schematise different flow paths present in the system. The main annulus flow consists of hot gas coming from the high-pressure turbine; the cooling flow supplied to the large inner cavity is colder air extracted from the compressor and delivered to the outer stator wells through appropriate holes.

The control points depicted in Figure 12 by black symbols represent the locations of several thermocouples used during engine testing. The metal temperatures recorded during the experimental survey are not directly available in this study. We can refer here to the results of a thermal model calibrated to match the measurements to within a small error. The thermal model of the turbine is a finite element model based on the Rolls-Royce proprietary code SC03 [32]. The thermal model, hereafter referred to as SC03 model, employs appropriately tuned heat transfer correlations to define the boundary conditions on the solid surfaces. The calibration in SC03 is carried out to match the available thermocouple data, that is, for a limited number of points. Hence, when looking at the details of the solution, the thermal model is not guaranteed to be correct away from the experimental measurement points.

Also note that the SC03 model is axisymmetric and that the blades over the discs are not included. Their effect on the thermal response is incorporated through the boundary conditions specified on the outer disc surfaces.

### 5.1. Computational Domain

With reference to Figure 12, the computational model adopted for this analysis includes three stator feet (SF1, SF2, and SF3), the underlying rotor discs (RD1, RD2, RD3, and RD4), and five fluid regions (three stator wells, the inner cavity, and the wheel space ahead of Stator Well 1). An attempt to include the main gas path into the analysis has been recently conducted by the same authors for a model limited to Stator Well 1 [31]. It was found that flow solutions based on RANS are unable to predict the correct amount of hot gas ingress and, as a result, the metal temperature within the cavity was strongly underpredicted. Ingress prediction is a complex, longstanding problem related to turbulence modelling [33,34,35] and is not the object of the current analysis. In this paper, inlet and outlet boundary conditions are applied at the rim seals of each stator well to guarantee the values of hot gas ingestion consistent with those prescribed in the benchmark SC03 model.

**Figure 13:**
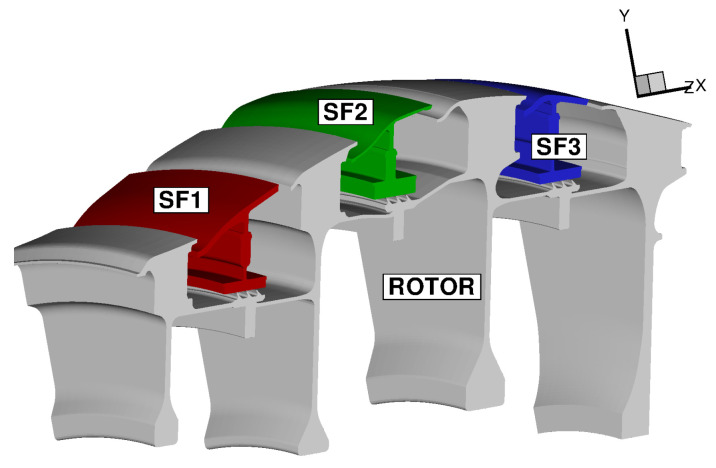
Solid models adopted for the low-pressure turbine.

**Figure 14:**
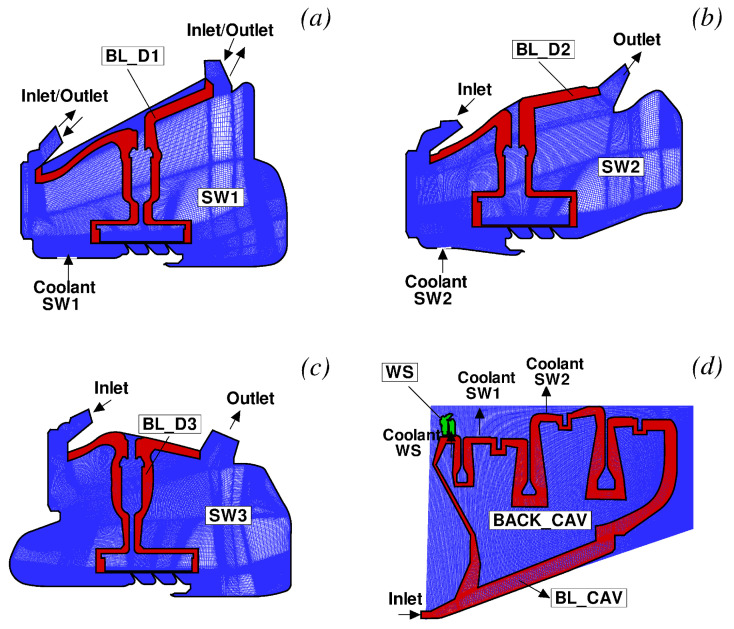
Fluid meshes adopted for the low-pressure turbine. (**a**) Stator Well 1; (**b**) Stator Well 2; (**c**) Stator Well 3; (**d**) Inner Cavity and Wheel Space.

**Figure 15:**
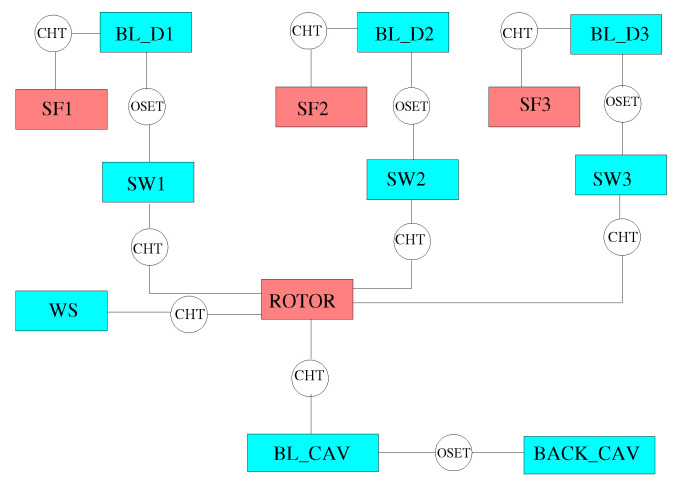
JMxx model of the low-pressure turbine. Refer to Figure 13 and Figure 14 for the nomenclature.

The solid models adopted for the study are shown in Figure 13. There are three disconnected domains for the stator feet and a single domain for the rotating part. The fluid zones have been meshed using overlapping grids, as shown in Figure 14. Each stator well is composed of a background mesh, constructed for a hollow cavity without any interior object, and an embedded boundary layer mesh built around the stator foot. Similarly, for the inner cavity (Figure 14d), a body-fitted mesh incorporating the features along the walls is contained within a Cartesian background mesh. This strategy facilitates preprocessing operations and can be conveniently adopted to replace a single component of the assembly without the need to reprocess the entire model. The final JMxx model is made up of 13 Hydra Sessions and 12 Coupler Units (see Figure 15). There is no direct link between the inner cavity and the outer stator wells. The coupling occurs through the solid model ROTOR, which is connected to the various fluid zones by CHT interfaces.

### 5.2. Model Set-Up

The SC03 thermal model of an engine component is built through laborious calibration work. Very briefly, the user specifies the mass flow rate, the fluid temperature, and the heat transfer coefficient for the flow streaming along each metal surface, and the wall temperature is then computed from one-dimensional energy budgets. The specifics for the SC03 model reflect the physical flow conditions developed in various parts of the component. This information is generally retrieved from standalone CFD simulations and 1D network flow models nested within an iterative multidisciplinary analysis [36,37].

**Figure 16:**
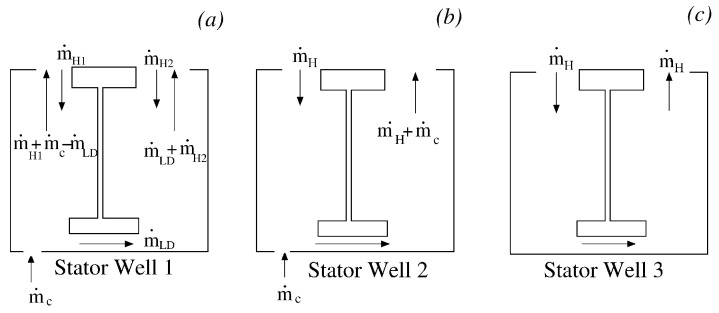
Cavity flow models assumed in the SC03 thermal model for the stator wells. (**a**) Stator Well 1; (**b**) Stator Well 2; (**c**) Stator Well 3.

The cavity flow models assumed in SC03 for the three stator wells are depicted in Figure 16. Stator Well 3 is without coolant. In this case, a certain amount of flow ${\dot{m}}_{H}$ penetrates the front cavity, moves through the labyrinth seal, and leaves the stator well from the rear cavity. In Stator Well 2, the coolant ${\dot{m}}_{c}$ is added to the ingested gas ${\dot{m}}_{H}$ before moving through the labyrinth seal. Stator Well 1 is characterised by egress conditions. In this case, the coolant is divided in two parts, with some of it moving into the labyrinth and the remainder leaving the cavity through the front rim seal after being mixed with the hot air coming from the annulus (${\dot{m}}_{H1}$).

The model set-up in JMxx needs to comply with the flow physics just described. The behaviour of Stator Well 2 and Stator Well 3 can be directly reproduced in the corresponding CFD models by specifying the mass flow rate, total temperature, and flow direction at the entry of the front cavity and for the cooling flow. The treatment of Stator Well 1 requires more attention. In fact, the schematic of Figure 16a represents the axisymmetric equivalent of a phenomenon that is strictly three-dimensional.

**Figure 17:**
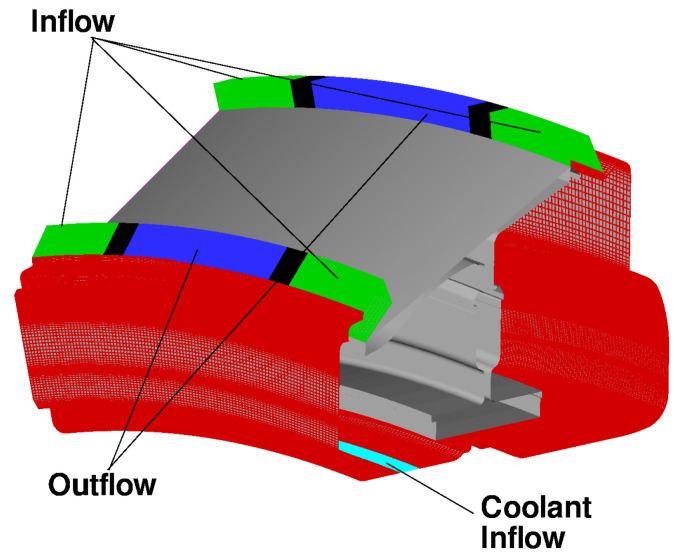
Inflow/outflow specification in the CFD model of Stator Well 1.

Even in conditions of net egress, the circumferential pressure variations that occur outside the cavity induce local ingestion in the regions with high pressure [34]. In order to reproduce this behaviour in the CFD model, the surfaces that define the rim seals of Stator Well 1 are split along the circumferential directions in three patches (see Figure 17), in which the boundary conditions are alternatively specified as inlet and outlet with fixed mass flow rate. More precisely, in accordance with the model of Figure 16a, for the inflow boundaries of the front and rear cavities (green surfaces in Figure 17), the mass flow rates are set equal to ${\dot{m}}_{H1}$ and ${\dot{m}}_{H2}$, respectively. For the outflow boundaries at the front and at the rear (blue surfaces in Figure 17), the mass flow rates are set equal to ${\dot{m}}_{H1}+\dot{m_{c}}-{\dot{m}}_{LD}$ and to ${\dot{m}}_{LD}+{\dot{m}}_{H2}$, respectively. The values of ${\dot{m}}_{H1},\;\dot{m_{c}},\;{\dot{m}}_{LD}$ and ${\dot{m}}_{H2}$ are taken equal to those specified in SC03. Thus, although the geometry has rotational symmetry, the boundary conditions are defined as three-dimensional to allow for consistent specification of the net amount of hot air passing through the cavities.

Table 2 summarises the model details for each fluid zone. The setting of the CFD method is heterogeneous. All models are steady RANS, and the implicit scheme, which is more efficient for flows dominated by diffusion, is adopted for the inner cavity. The choice of the turbulence model is the result of several numerical experiments aimed at improving the agreement with the metal temperature predicted by SC03. In this regard, it is important to remark that, while a single case initialised with uniform conditions and running on 400 cores required about one week to converge, each subsequent adjustment could be carried out in a single day. The resources allocated to handle the interfaces are 12 in total, one for each Coupler Unit.

The test case corresponds to the cruise conditions of an engine currently in service. For this reason, temperature values and mass flow rate ratios cannot be reported. Hereafter, all temperatures are expressed as $T^{*}=\left(T-T_{c}\right)/\left(T_{H}-T_{c}\right)$, where $T_{c}$ is the total temperature at the entry of the inner cavity, and $T_{H}$ is the total temperature of the annulus flow entering Stator Well 1. The rotational Reynolds number, based on the rotor angular speed, the radius at point P4 (Figure 12), and the flow conditions in the rim seal at the front of Stator Well 1, is equal to $Re_{\theta }=2.3\times 10^{5}$.

### 5.3. Results

**Figure 18:**
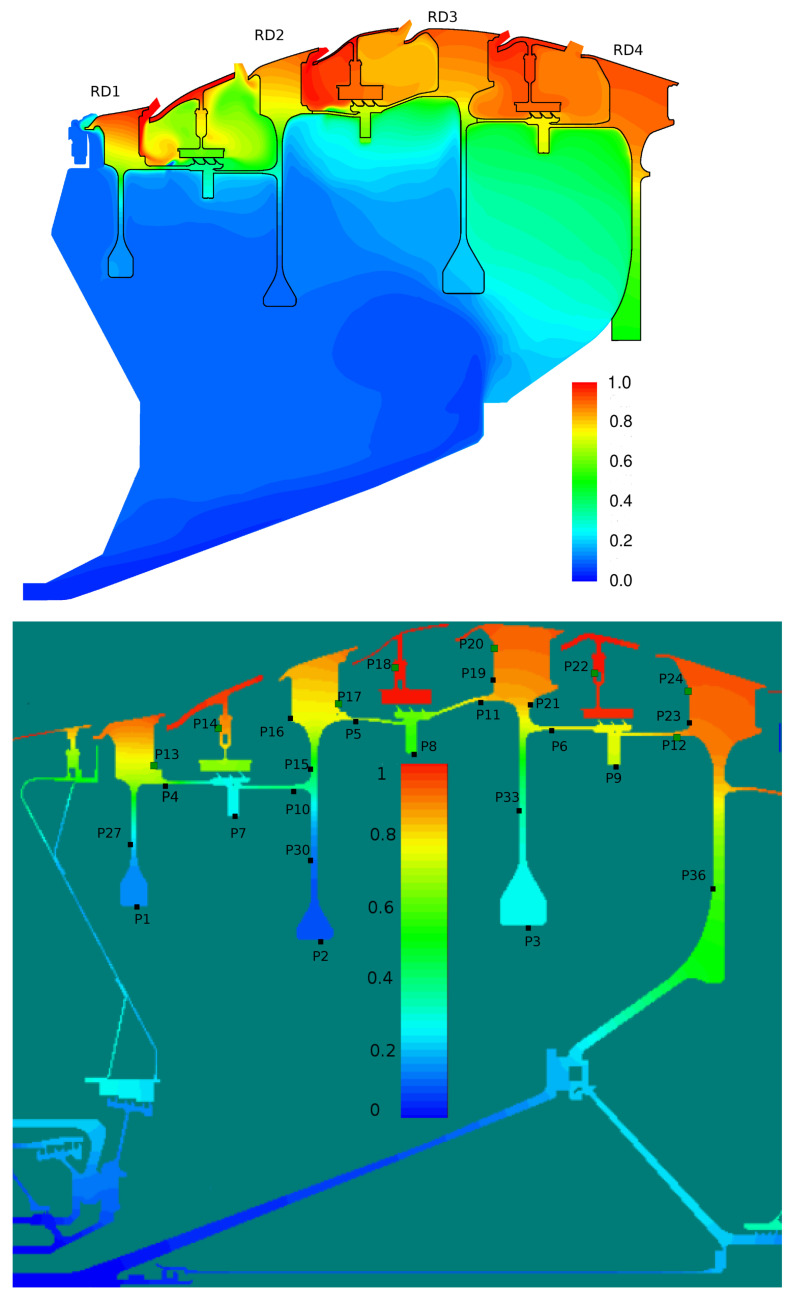
Nondimensional temperature predicted by JMxx (**top**) and by SC03 (**bottom**). Refer to Figure 12 for control points from P25 to P35.

Figure 18 shows the coupled temperature field computed by JMxx and the solid temperature predicted by SC03. A good qualitative agreement between the two solutions is achieved, and it is possible to correlate the thermal response of the metal with the observed flow behaviour. The largest amount of coolant is supplied to Stator Well 1. Here, the stator foot, which is heated on the outer surface from the annulus flow, shows a strong thermal gradient in the radial direction due to the presence of coolant that fills both the front and the rear cavity. The amount of coolant introduced in Stator Well 2 is relatively smaller, and the jet with cold air appears to be all sucked into the labyrinth seal. As a result, the temperature field shows a marked discontinuity between the front and the rear cavities. Stator Well 3 is without cooling flow. Both front and rear cavity are filled with hot gas, and the temperature distribution is more uniform.

**Figure 19:**
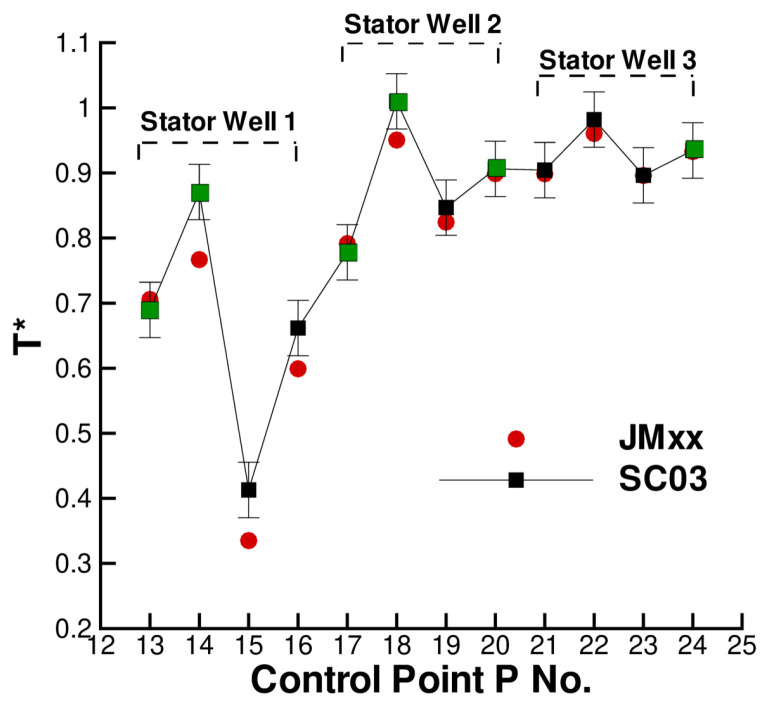
Temperature at the control points within the stator wells. Black squares correspond to thermocouple locations.

**Figure 20:**
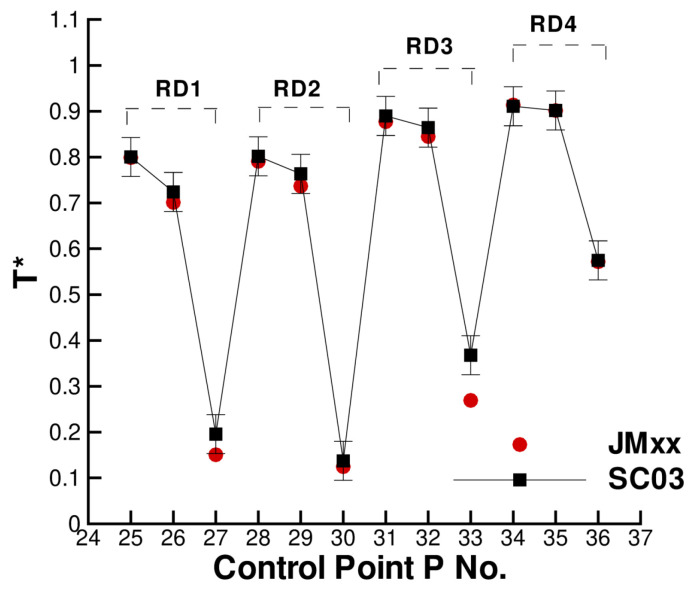
Temperature at the control points on the rotor discs. Black squares correspond to thermocouple locations.

A more quantitative comparison between JMxx and SC03 thermal predictions is given in Figure 19 and Figure 20. Here, we report the temperature predicted at several points located inside the stator wells and along the discs. Only the monitor points denoted by black symbols correspond to a thermocouple, and these are all located on rotating components. Green symbols are additional control points selected for the current analysis. Note that the error bars used in Figure 19 correspond to the *overall* accuracy reported for the thermal model. More specific information concerning the error at each control point is not available. There is a good agreement between the two solutions, especially for the points inside Stator Well 2 and Stator Well 3. In Stator Well 1, the agreement is less satisfactory, although the trend is well captured. The largest error (10%) occurs at point P14 in the central part of the stator foot. With the help of Figure 18, it can be recognised that the discrepancy becomes smaller in the upper and lower parts of the component. For the points located in the upper part of the rotor discs, there is a substantial agreement with the measurements. At inner radii, point P33 on disc RD3 can be noted, where the temperature is significantly underpredicted. This discrepancy is examined later.

**Figure 21:**
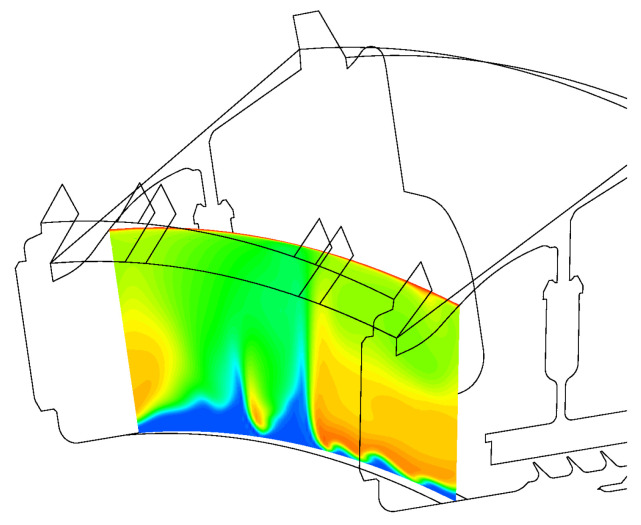
Temperature contour plot on a cross section of Stator Well 1.

Figure 21 shows the fluid temperature on a cross section of Stator Well 1. The figure reflects the mixed specification of inlet/outlet boundaries, highlighting a central region where the coolant penetrates deeper into the cavity. It is understood that some uncertainties in the model specification remain. First, the strong interaction between the coolant and the ingested flow is unsteady, and although the solid temperature converges well to a mean value, unsteady effects may not be well captured by the steady RANS solver. In addition, the number of patches selected for the entry surface of the stator well may have an impact on the mixing process.

**Figure 22:**
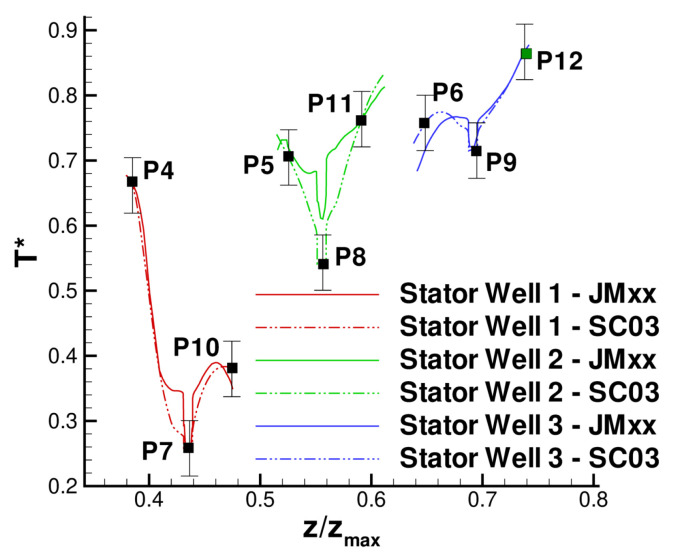
Axial temperature profiles on the upper surface of the inner cavity. Black symbols correspond to thermocouple locations.

**Figure 23:**
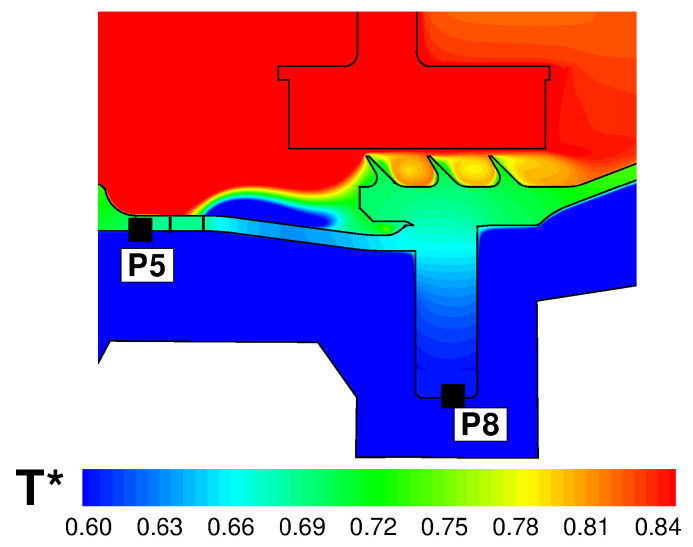
Close-up view of the temperature field in the labyrinth seal of Stator Well 2.

On the basis of these uncertainties, the thermal prediction of JMxx can be deemed reasonably good. Figure 22 shows axial temperature profiles retrieved for the upper surfaces of the inner cavity. For the majority of the control points, there is a close agreement with the SC03 solution, and the profiles show a certain similarity. The JMxx solutions present “jumps” in the temperature profiles, occurring in the metal protrusions that extend into the domain of the Inner Cavity. These jumps are stronger for Stator Well 1 and Stator Well 2, suggesting a connection with the presence of coolant. To examine the behaviour, it is convenient to focus on Stator Well 2, where the inlet/outlet conditions are axisymmetric and the flow conditions are more regular than in Stator Well 1. Figure 23 shows the temperature field in the labyrinth seal. It can be seen that, in the small cavities between the labyrinth fins, the air temperature is significantly higher than the inlet temperature of the coolant. This indicates that a considerable amount of hot flow is mixed with the cooling flow. Note also, on the right of the secondary inlet, a confined region with cold fluid, that keeps the underlying metal at low temperature. In the labyrinth seal, the acceleration imposed on the flow by the constrictions enhances heat transfer by convection, and the metal fins, which have small thermal capacity, are heated up. Since the solid protrusion is cooled on the inner side, heat is transferred radially inwards by conduction, and the metal temperature reaches a minimum at point P8. The jumps observed in Figure 22 identify radial temperature gradients for the boundary points located on the two parallel sides of the protrusion. In Stator Well 3, this mechanism occurs on a smaller scale due to the absence of the secondary flow. It can be seen in Figure 18 that the solid protrusion is wet on the inner side by a fluid region with little recirculation, where a smaller amount of heat is extracted from the solid.

In the SC03 prediction, the jumps are not completely absent, but they are substantially smaller. The reason for this is that the temperature distribution in the labyrinth seal regions is much more uniform (see Figure 18). In this regard, the SC03 solution is less convincing, as it indicates an equal fluid temperature on the inner and outer sides of the protrusion or, equivalently, that the flow moving through the labyrinths in Stator Well 1 and Stator Well 2 consists predominantly of cold fluid. This is contrast with the behaviour observed in Figure 23. As a further remark related to these arguments, it is possible to state that, owing to the radial extent of the solid protrusion, the mismatch with the thermocouple measurement at point P8 (Figure 22) is probably associated with the behaviour of the flow in the zone Inner Cavity, as also suggested in the considerations that follow.

**Figure 24:**
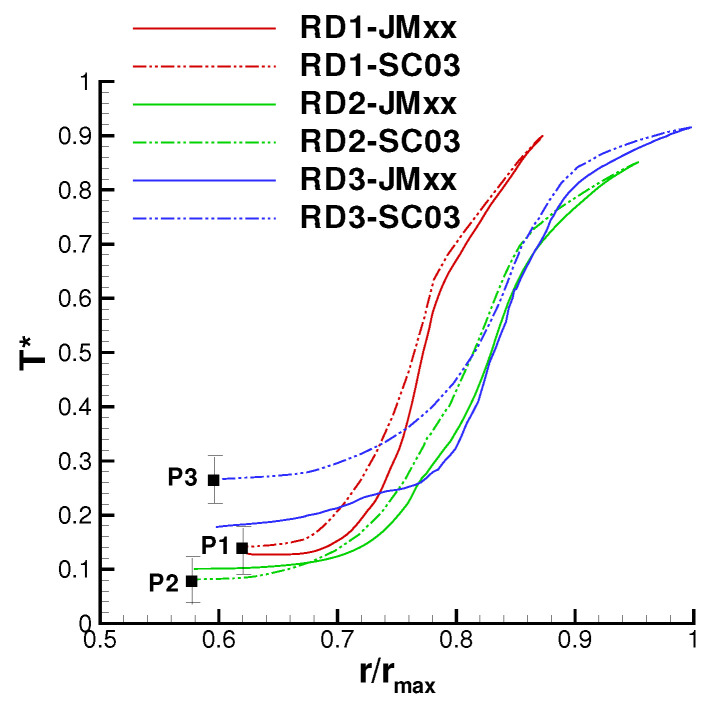
Radial temperature profiles along the rotor discs. Black symbols correspond to thermocouple locations.

The temperature profiles along the rotor discs are given in Figure 24. The behaviour of disc RD1 and disc RD2 is well captured, and the agreement at the inner tips of the discs (points P1 and P2) is very good. The temperature profile for RD3 is significantly different. It can be noted in Figure 18 that RD3 separates a cavity where cold fluid enters from inner radii and recirculates (on the left of RD3), from a cavity with hotter fluid where very little convection occurs (on the right of RD3). It is possible that, in the CFD solution, less flow is diverted towards the cavity on the left of RD3 and a small amount of coolant remains channelled in the cavity on the right. This may also explain the overprediction observed at point P8 in Figure 22. In our tests, we noticed a certain sensitivity of the resulting flow field to the selected turbulence model. The overall impression is that some mechanisms (buoyancy and mixing) may not be properly captured by RANS and may require a higher fidelity approach (LES).

## 6. Scalability Analysis

The aim of this section is to describe the main scalability features of JMxx. For this purpose, two different tests are considered. In a first one, we focus on the concurrency between search operations of CU processes and internal iterations of HS processes (see Section 3.3) and illustrate how the overhead for handling an interface can be made scalable by splitting the interface, as described in Section 3.2. In a second test, we measure strong scalability for a URANS computation performed on a simple (but realistic) model involving a sliding plane.

### 6.1. Concurrency Tests between HS and CU Precesses

**Figure 25:**
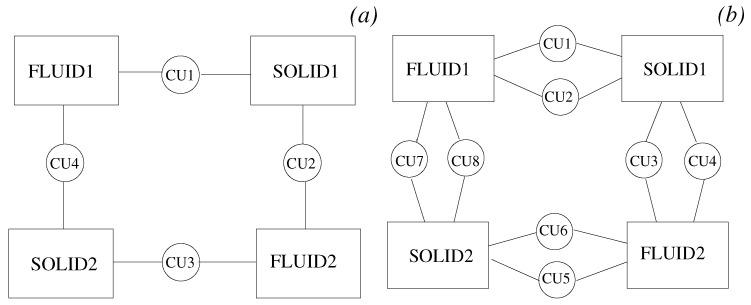
JMxx models used for the concurrency test. (**a**) Model A, with one interface for each coupled surface; (**b**) Model B, with two interfaces for each coupled surface.

We consider a problem where two solid domains are coupled with two fluid domains according to the schematic of Figure 25. Each domain is a cube, and the corresponding mesh is a structured block discretised by $n_{x}=4\times 10^{2},n_{y}=4\times 10^{2}$, and $n_{z}=10$ nodes in the *x*, *y*, and *z* direction, respectively. The overall assembly has 6.4 million mesh nodes and four coupled surfaces with $4\times 10^{3}$ nodes each. Two JMxx models are considered for the same problem. Model A, shown in Figure 25a, includes 4 Coupler Units; in Model B, the surfaces between the domains are split in two portions of equal size and the corresponding JMxx model has a total of 8 Coupler Units, (Figure 25b).

The coupling parameters in JMxx are intentionally set to trigger the conditions under which the scalability of the coupler deteriorates. The exchange of data and the element containment search is performed at the end of any iteration, and a “brute force” sequential search is carried out by looping for each target node over the entire set of source elements that lie on a single interface. The containment test includes a projection onto each of the four triangles forming the source element, followed by inversion of four linear systems of size 2×2 to determine the natural coordinates of the projected point. The spatial search can be made faster by orders of magnitude using bounding boxes, i.e., limiting the operations to the source elements that lie within a small distance from the target nodes. In the present test, bounding boxes are turned off. In other words, the test is designed to increase the workload of the CU processes to the point where it is comparable to that of the HS processes. In this way, we reproduce on a small model the conditions that, in a larger model, are responsible for a performance decay if running on several thousands of processes. In such a pathological condition, we analyse the effect of increasing the number of processes per Coupler Unit and the effect of splitting the interfaces.

**Figure 26:**
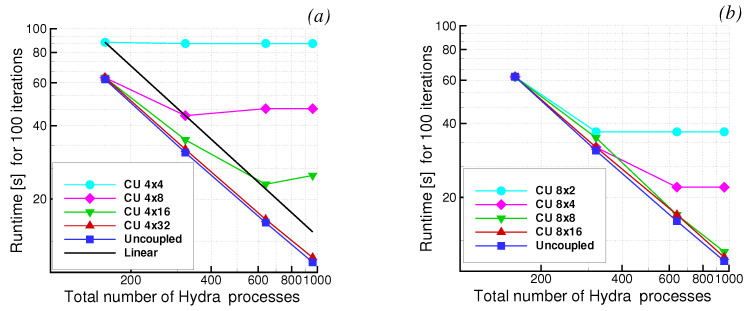
Scalability analysis for the concurrency test. (**a**) Results obtained for Model A; (**b**) Results obtained for Model B.

The tests were conducted on a Cray CS-400 system equipped with Intel Xeon E5-2600 CPUs of 24 cores and a Mellanox Infiniband high-speed interconnect. Figure 26 shows the execution time measured for 100 iterations. The total number of cores allocated for the Hydra Sessions is equally distributed among the zones and gradually increased from 160 to 320, 640, and 960. The various curves in Figure 26 correspond to a different number of cores allocated (and equally distributed) for the Coupler Units. The behaviour of JMxx is assessed with reference to an uncoupled simulation conducted for the same model, with Hydra having all of the communication routines between the HS and CU processes turned off. The uncoupled simulation does not resolve the fluid/solid problem and is adopted here to report the scaling behaviour associated with the internal parallisation of Hydra.

Using four cores for each Coupler Unit, the execution time of Model A is insensitive to any variation in the number of HS processes (Figure 26a). In this case, most of the work is associated with the search operations, and the HS processes are sitting idle, waiting for CU processes to complete. As the number of CU processes is doubled (4×8) the search operations are sped up, and the performance of the uncoupled model is recovered when 160 Hydra cores are used. The corresponding curve lowers and remains almost constant after 320 HS cores. By gradually increasing the number of CU cores, the process continues: the number of Hydra cores where the various curves flatten becomes gradually higher. These “breakdown points” mark the transition between the case in which Hydra iterations are faster than coupling operations (HSs wait for CUs) and the opposite condition (CUs wait for Hydra iterations to finish). The linear scaling, identified in the figure by the black solid line, intersects the breakdown points of the curves almost exactly. This indicates that the workload of the Coupler Units scales linearly. When 4×32 cores are used, the curve finally collapses into that of the uncoupled model, with a slight degradation presumably due to the communications. Thus, as long as the search is quick enough to be “hidden” within a single Hydra iteration, the coupled model scales as its Hydra components.

The results of Model B (Figure 26b) reflect the obvious behaviour of a parallel implementation search. With the same amount of resources allocated for the Coupler Units, Model B is faster than Model A, and less CU processes are needed in order to recover the performance of the uncoupled model. The execution time of the search algorithm (with bounding boxes turned off) scales as $N_{t}\times N_{s}$ where $N_{t}$ is the number of target nodes and $N_{s}$ the number of source elements (in our example $N_{t}≃N_{s}$). Clearly, doubling the CU processes corresponds to a reduction by a factor of 2 in Model A and by a factor of 4 in Model B. The present results illustrate the opportunity to offload and schedule a search and interpolation workload earlier, overlapping it with the main computation.

It is worth noting that parallelisation of the communications, which in a larger model are certainly more relevant, follows the same logic and can equally benefit from the strategy of splitting the interfaces.

### 6.2. Case Study with Sliding Plane

Sliding planes are a common method used in turbomachinery to handle the unsteady coupling between rotating components in URANS simulations. In Hydra, stator and rotor passages are resolved in their own frame of reference and joined together using overlapping cells at the interface to provide a second-order discretisation scheme. Due to their nature, sliding planes are typically difficult to balance and optimise for parallel efficiency and, in large multi-row 360 deg models, very quickly become the main bottle-neck for the speed-up of the simulation.

**Figure 27:**
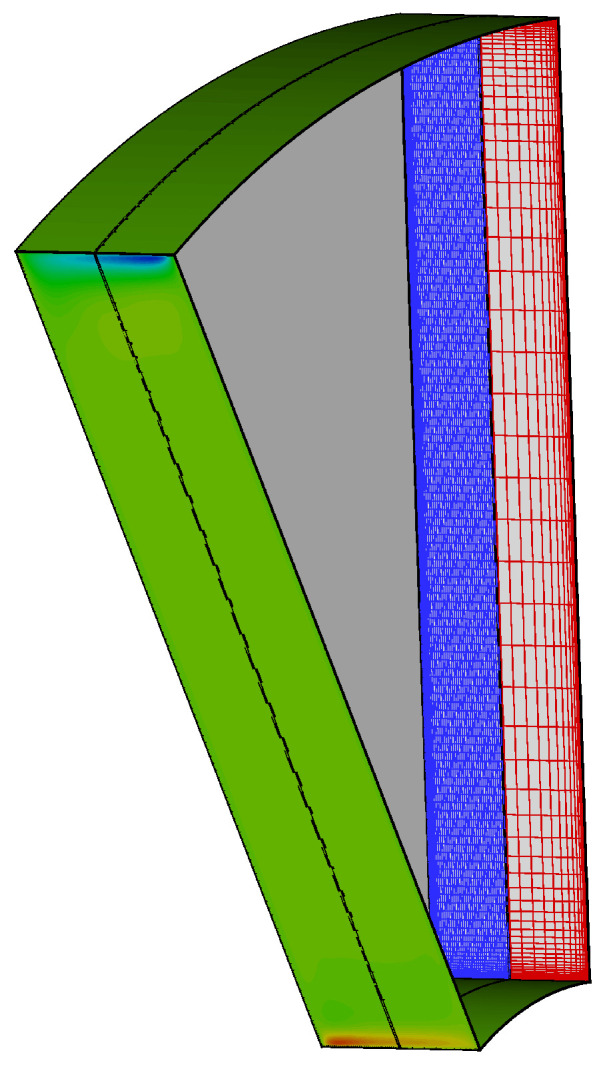
Rotor-stator cavity model adopted for the sliding plane test. Axial velocity contours are shown on the periodic surface.

As demonstrated in the previous example, one way to improve the parallelism is to enforce geometric conditions in the construction of the interface, so that the search performed at each time step can be more efficient. This type of tuning fits naturally in the capability of the coupling approach proposed and different strategies can be easily implemented. As a more realistic example, the results for a 30${}^{\circ }$ sector model of a rotor-stator cavity are shown herein (Figure 27). The cavity is formed by two fluid zones, both solved as URANS. The stationary domain (blue mesh in Figure 27) is separated from a rotating domain (red mesh) by a sliding plane located at the cavity centre. The stator mesh consists of 924 thousand nodes, of which 21 thousand are on the sliding plane with a relative ratio equal of 1:44. The rotor mesh has 67.2 thousand mesh nodes and 4200 sliding plane nodes (relative ratio of 1:16).

The tests were executed without multigrid using Dual Time Stepping, with the number of inner iterations equal to ncycle=25 for each physical time step. The Hydra processes were gradually increased from 44 (corresponding to an average number of mesh nodes per process $N_{ave}≃23,000$ to 704 $\left(N_{ave}≃1400\right)$, maintaining load balancing between the two Hydra Sessions. The sliding plane is divided into an increasing number of radial bands (from 1 to 8) containing a very similar number of nodes. Each radial band corresponds to a Coupler Unit, and each Coupler Unit has one MPI rank. The method used to pinpoint an appropriate distribution of the radial bands is described in Appendix B. It is important to note that the memory requirements of the coupler units scale perfectly linearly with the portion of the grid interacting through the coupled interface.

**Figure 28:**
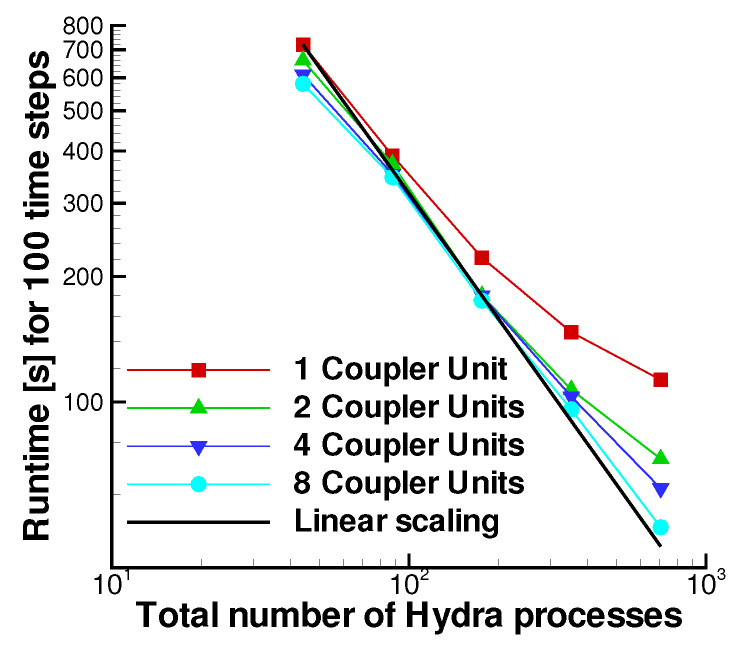
Scalability analysis for the rotor-stator cavity model.

Figure 28 depicts strong scaling of the coupled application in terms of the runtime of 100 time steps. From that, it is possible to see that the model scales increasingly better up to 704 cores, exhibiting the strong scalability limits of a typical CFD finite volume based monolithic code. Despite being a simple model, the overhead of the sliding plane, which is measured by the ratio between mesh nodes and sliding plane nodes, is very demanding. The test shows that, with a proper coupling logic based on geometrical constraints and with a flexible use of the available computing ranks dedicated to the solver or the Coupler Units, linear scaling can be satisfactorily recovered. It is finally worth mentioning that the implementation of this as well as different improved logic occurs inside the coupling sliding-plane kernel and therefore, it does not affect the coupler framework. Additionally, being at the same time outside of the solver, it does not require any update or change either.

## 7. Conclusions

In this paper, we have presented a high-performance inter-code coupling framework for distributed execution of coupled multi-physics solvers using the suite of CFD Hydra codes as an example. Conceptually, the proposed JMxx framework avoids the creation of a centralised communication hub by launching as many distributed instances of the same coupler abstraction as there are coupled interfaces. The Coupler Units are employed as individual servers for each interacting interface between the coupled models. The selected flexible modular approach enables effortless point-to-point inter-code communication.

Several typical turbomachinery analysis scenarios have been discussed, involving moving and stationary fluid–fluid, fluid–solid, and solid–solid interfaces. Parallel performance of the coupling framework was assessed using the time to solution and the strong scaling indicators. The performance results show that JMxx can recover nearly linear scalability of a monolithic single-physics Hydra CFD solver.

Although the JMxx coupler abstraction was designed primarily for turbomachinery flows, the system architecture and many of the underlying concepts have a broader range of applicability in scientific simulations codes involving time stepping systems. We believe that the main advantage of JMxx is in its conceptual simplicity and non-intrusiveness: by enforcing a direct correspondence between the Coupler Unit and interface, the coupler can be viewed by an individual code as another type of boundary condition, regardless of the complexity of the overall coupled system behind it and without any scheduling burden.

Work is currently in progress to equip JMxx with multi-scale capability, enabling time-scale separation and introducing appropriate interfaces for hybrid modelling involving LES, steady, and unsteady RANS. In this context, more work will also be needed to develop a system for automatic allocation of resources. This will serve to optimise static load-balancing of computations and communication work between individual codes and coupler units in a heterogeneous system.

Although this may not be a simple task, the architecture of JMxx offers a natural advantage for the purpose: treating the interfaces separately, the overhead for handling each interface can be made scalable by paying a small penalty, namely having a tiny percentage of resources (those in charge of the CUs) running faster than the rest. Thus, if we are also able to guarantee the local balance described by Equation (1) for any type of interface, regardless of mesh type/size, physical model, discretisation scheme, etc., a global solution would always be available enforcing Equation (1) for all Coupler Units. The resulting distribution of resources would be suboptimal either in the least squares sense or in the sense of Lagrange multipliers.

## Figures and Tables

**Table 1:** Representative sections of the data structures used in JMxx: Coupler Table (**top**) and Hydra Table (**bottom**).

STRUCTURE coupler_table	
**string** ctype	Interface type
**integer** cproc	Number of cores for the CU
**integer** hsession(2)	Session id of attached HSs
**integer** hproc(2)	Number of cores for attached HSs
⋯	
**integer** rank_h(hproc,2)	Partition ranks of attached HSs
⋯	
**integer** ntnode(hproc,2)	Number of target nodes on the interface
⋯	
**real** rot(2)	Zonal rotational speed
⋯	
**pointer** p_xtarget(2)	Memory address of target nodes
**pointer** p_xsource(2)	Memory address of source nodes
**END STRUCTURE**	
**STRUCTURE hydra_table**	
**string** ctype(ncoupl)	Interface type
**integer** hproc	Number of cores for the HS
**integer** cproc(ncoupl)	Number of cores for attached CUs
⋯	
**integer** rank_c(cproc,ncoupl)	Partition ranks of attached CUs
⋯	
**integer** ntnode(cproc,ncoupl)	Number of target nodes on the interface
⋯	
**pointer** p_xtarget(cproc,ncoupl)	Memory address of target nodes
**pointer** p_xsource(cproc,ncoupl)	Memory address of source nodes
**END STRUCTURE**	

**Table 2:** Turbulence model (SST: Kω-SST, SA: Spalart-Allmaras, EARSM: Explicit Algebraic Reynolds Stress Model). Time integration method (exp: explicit multigrid scheme, imp: implicit scheme on single grid). Mesh size in million nodes of each fluid zone.

	SW1	BL_SF1	SW2	BL_SF2	SW3	BL_SF3	WS	BL_CAV	BACK_CAV
Model	SST	SST	SA	SA	SA	SA	SA	EARSM	EARSM
Scheme	exp	exp	exp	exp	exp	exp	exp	imp	imp
Size	7.4	3.0	7.3	3.5	8.0	3.7	1.6	10.3	7.3

**Figure A1:**
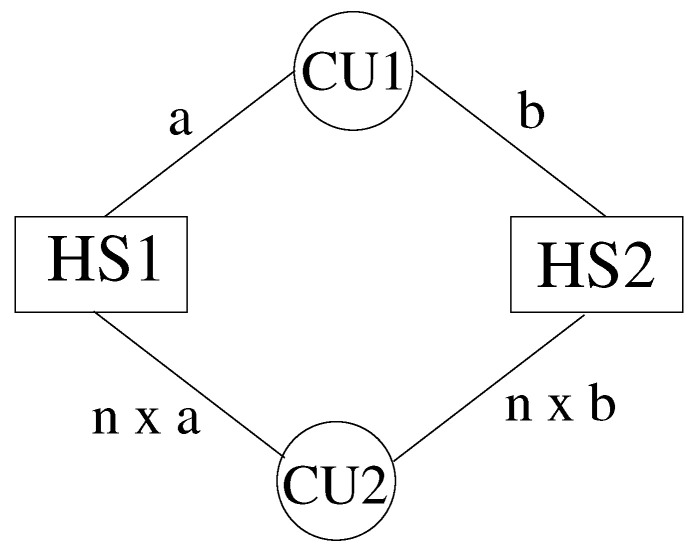
Definition of a Solvable Elementary Cycle.

**Figure A2:**
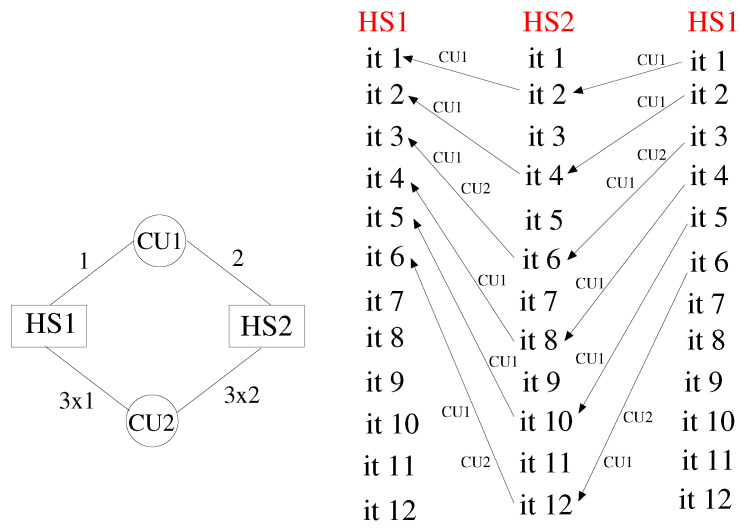
Solvable Elementary Cycle with a=1,b=2,and n=3.

**Figure A3:**
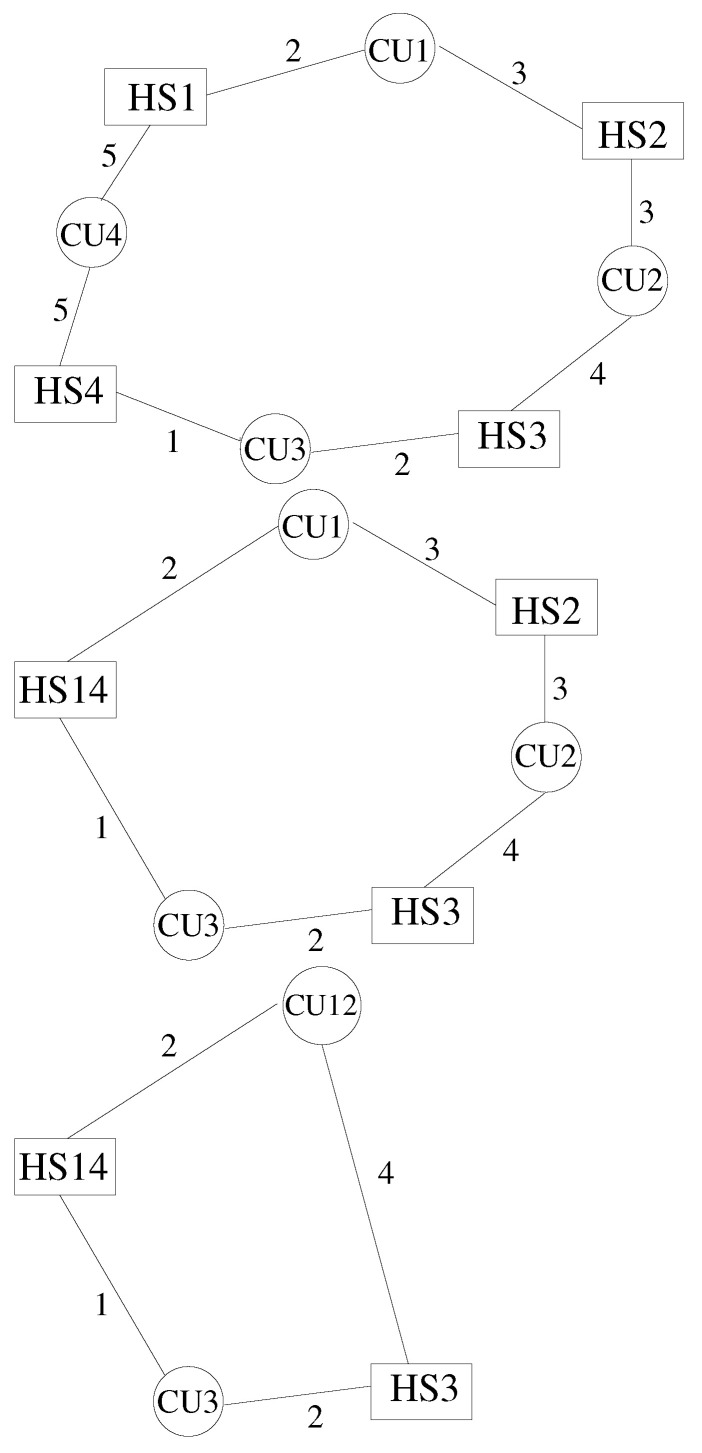
Simple method for deadlock detection in a JMxx cyclic model (**top**). At the first step, HS1 and HS4 are collapsed into HS14 (**center**). At the second step, CU1 and CU2 are collapsed into CU12 (**bottom**). The model is deadlock free because the corresponding graph is reduced to that of a Solvable Elementary Cycle.

## Data Availability

Additional data have not been reported.

## Appendix A. Deadlock Avoidance

A Solvable Elementary Cycle is defined as a JMxx cyclic model with two HSs; two CUs; and coupling frequencies a,b,n×a, and n×b (Figure A1). Using dependency patterns, it is easy to see that a Solvable Elementary Cycle can never incur a deadlock, regardless of the value assumed by *n*. An example is reported in Figure A2 for a=1,b=2, and n=3. Coupler Unit CU1 receives communication requests for every single iteration from HS1 and every two iterations from HS2, while the requests received from CU2 occur at iteration numbers that are the same multiple of *a* and *b*. This guarantees that the synchronised mutual dependency is preserved.

For a cyclic model with an arbitrary number of HSs and CUs, a sufficient condition to avoid deadlock can be derived with the help of transparent CUs and transparent HSs. A Coupler Unit is said to be transparent if it has equal coupling frequencies on both sides. For an Hydra Session, the definition of transparency is related to interfaces belonging to the same cycle. Thus, an Hydra Session is transparent with respect to a certain cycle if the coupling frequencies on the two interfaces lying on the cycle are equal.

Transparent CUs and transparent HSs have the property of not affecting the synchronisation of a cycle. In other words, a model obtained by enlarging a Solvable Elementary Cycle with an arbitrary number of transparent CUs and HSs, each with its own coupling frequency, is deadlock free. With the same arguments, it is possible to state that, if the graph of a cyclic model can be reduced to that of a Solvable Elementary Cycle through removals of transparent CUs and transparent HSs, the model is deadlock free. Figure A3 illustrates the concept with an example. The JMxx model includes one transparent CU (CU4) and one transparent HS (HS2), which can be both removed from the graph. The removal of CU4 means that HS1 and HS4 are collapsed into HS14. At the successive step, CU1 and CU2 are collapsed into C12 for the removal of HS2. The reduced graph corresponds to that of a Solvable Elementary Cycle. Hence, the model is deadlock free. If more cycles are present in the model, the above condition should be verified for each of them.

## Appendix B. Surface Splitter Emulator

The radial bands, or other types of geometric constraints adopted to split the interface, are specified in the input file of JMxx. A small preprocessing tool helps the user in this task. For any surface defined in the mesh, it is possible to identify a specific direction along which the nodal distribution can be parallelised. Obviously, if a relative movement is involved, it is necessary that the nodes belonging to the different patches remain confined in the same spatial region. For the sliding plane of a turbine stage, the obvious choice is the radial direction, but if the interface extends along another direction, different choices would be more appropriate.

Thus, without loss of generality, we can assume a nodal distribution F=F(r) along the selected direction *r*, with F(r) varying between 0 and the total number of nodes *N* that lie on the interface. In the method, a cubic spline interpolation is first determined for F(r), and a balanced choice of the bands is obtained by solving the equation

(A1) $$\begin{array}{c} F\left(r_{2}\right)-F\left(r_{1}\right)=N/n \end{array}$$

where *n* is the prescribed number of bands and $r_{1},r_{2}$ are values ranging from $r_{min}$ and $r_{max}$, which are iteratively updated as shown in the following steps:

1. $r_{1}=r_{min}\;\;;\;\;F\left(r_{min}\right)=0$
2. Solve $F\left(r_{2}\right)=N/n+F\left(r_{1}\right)$
3. $r_{1}\leftarrow r_{2}$
4. Go to step 2 or exit if $r_{2}=r_{max}$

Best practice rules based on the ratio between interface nodes and mesh nodes guide the user to select the value of *n*, which allows for sufficient scalability of the interface.
